# Recurrent Takotsubo Cardiomyopathy With Variable Patterns and Psychiatric Comorbidities: A Case Report and Comprehensive Literature Review

**DOI:** 10.1002/ccr3.70635

**Published:** 2025-07-10

**Authors:** Hamza AlKowatli, Oubada Alkowatli, Ahmad Karzoun, Bisher Sawaf, Mhd Baraa Habib, Nahush Bansal, Muhammad Faiz, Robert Subbiondo

**Affiliations:** ^1^ Department of Internal Medicine HCA Blake Hospital Bradenton Florida USA; ^2^ Department of Internal Medicine Endeavor Health Chicago Illinois USA; ^3^ Department of Internal Medicine University of Toledo Toledo Ohio USA; ^4^ Department of Cardiology, Heart Hospital Hamad Medical Corporation Doha Qatar

**Keywords:** psychiatric comorbidity, recurrence, stress‐induced cardiomyopathy, Takotsubo cardiomyopathy, wall‐motion variants

## Abstract

Takotsubo cardiomyopathy (TTC) is a transient stress‐induced cardiac syndrome that can mimic acute coronary syndrome but typically lacks obstructive coronary disease. We report a case of a 50‐year‐old woman with complex psychiatric comorbidities who developed classical apical TTC following acute emotional distress—distinct from a prior reverse TTC episode during medical illness. Her condition stabilized with supportive care, anticoagulation, and psychiatric management. This case illustrates the phenotypic variability of TTC, the role of emotional stress in recurrence, and the importance of integrated multidisciplinary care.

**Summary**

- Recurrent Takotsubo cardiomyopathy may present with different wall‐motion patterns and is often triggered by emotional stress, particularly in patients with psychiatric illness.
- Recognition, imaging‐based diagnosis, and coordinated psychiatric and cardiac care are essential for effective management and recurrence prevention.

**Abbreviations**
ACE: angiotensin‐converting enzyme
ACS: acute coronary syndrome
ARB: angiotensin receptor blocker
BB: beta‐blocker
BNP: B‐type natriuretic peptide
CAD: coronary artery disease
CBT: cognitive behavioral therapy
CK‐MB: creatine kinase‐MB
CMR: cardiac magnetic resonance
CO₂: carbon dioxide
COPD: chronic obstructive pulmonary disease
CT: computed tomography
CTA: computed tomography angiography
DM: diabetes mellitus
ECG: electrocardiogram
EEG: electroencephalogram
EF: ejection fraction
GAD: generalized anxiety disorder
GERD: gastroesophageal reflux disease
hs‐TnT: high‐sensitivity troponin T
HTN: hypertension
ICD: implantable cardioverter‐defibrillator
ICU: intensive care unit
LAD: left anterior descending
LV: left ventricle
LVEF: left ventricular ejection fraction
LVOT: left ventricular outflow tract
MDD: major depressive disorder
MRA: mineralocorticoid receptor antagonist
MRI: magnetic resonance imaging
NSTEMI: Non‐ST elevation myocardial infarction
NT‐proBNP: N‐terminal pro B‐type natriuretic peptide
PE: pulmonary embolism
pH: potential of hydrogen
QTc: corrected QT interval
SSc: systemic sclerosis
SSRI: Selective Serotonin Reuptake Inhibitor
THC: tetrahydrocannabinol
Tn‐T: troponin T
TTC: takotsubo cardiomyopathy
TTE: transthoracic echocardiogram
VF: ventricular fibrillation
WBC: white blood cell

## 1 Introduction

Takotsubo cardiomyopathy (TTC), also known as stress‐induced cardiomyopathy or broken heart syndrome, is a transient cardiac condition characterized by acute left ventricular dysfunction, often mimicking acute coronary syndrome (ACS) in its presentation. First described in Japan in 1990, the syndrome derives its name from the Japanese word “takotsubo,” which refers to an octopus trap that resembles the shape of the left ventricle during the acute phase of the condition [1, 2]. The pathophysiology of TTC is not fully understood, but it is believed to involve a surge in catecholamines triggered by physical or emotional stress, leading to myocardial stunning and transient left ventricular dysfunction. Other proposed mechanisms include coronary vasospasm, microvascular dysfunction, and direct catecholamine‐mediated myocardial injury [3]. The condition predominantly affects postmenopausal women, with a mean age of 65 years, and is often precipitated by a significant emotional or physical stressor [2, 4].

Clinically, TTC presents with symptoms similar to those of ACS, including chest pain, dyspnea, and electrocardiographic changes such as ST‐segment elevation or T‐wave inversion. However, unlike ACS, coronary angiography in TTC typically reveals non‐obstructive coronary artery disease. Echocardiography is crucial for diagnosis, demonstrating characteristic left ventricular apical ballooning with basal hyperkinesis [5, 6]. Recurrent TTC is a recognized phenomenon, although it is relatively rare. Studies have reported recurrence rates ranging from 1.3% to 9.1% over varying follow‐up periods [1, 5]. Factors associated with recurrence include the absence of an identifiable trigger, physical stressors, chronic obstructive pulmonary disease, and a history of depression [1, 5]. The recurrence of TTC can present with different morphological patterns, as highlighted in case reports where patients exhibited both classical and reverse TTC during separate episodes [5].

Management of TTC primarily involves supportive care, with the use of heart failure medications such as angiotensin‐converting enzyme inhibitors and angiotensin receptor blockers showing potential benefits in reducing mortality and recurrence rates [3]. Beta‐blockers, although commonly used, have shown controversial results regarding their efficacy in preventing recurrence [3]. In cases complicated by left ventricular outflow tract obstruction, beta‐blockers and fluid administration are recommended, while inotropes are preferred over vasopressors in other complicated cases [3]. Given the potential for recurrence and the associated morbidity, long‐term follow‐up and management of predisposing factors are essential. Further research is needed to better understand the pathophysiology of TTC and to develop evidence‐based guidelines for its management, as current recommendations are largely based on expert opinion and retrospective analyses [7].

## 2 Case Presentation

### 2.1 Initial Presentation

A 50‐year‐old woman presented to the emergency department with acute‐onset gastrointestinal and cardiopulmonary symptoms, including nausea, vomiting, abdominal pain, intermittent chest pain, dyspnea, and escalating anxiety that had persisted over several days. According to her husband, she exhibited increasing paranoia and agitation, repeatedly expressing fear of an imminent heart attack. On initial evaluation, she was alert and oriented; however, her condition rapidly deteriorated. She became acutely confused, agitated, and combative, with incoherent speech, flailing limb movements, and an inability to follow simple commands or recall basic personal details such as her age or the current month. Given these concerns, a stroke alert was activated.

Vital signs at presentation showed elevated blood pressure and heart rate, along with mild tachypnea. The patient was afebrile and maintained oxygen saturation on room air. Physical examination revealed fluctuating levels of alertness, disorganized speech, and profound disorientation, consistent with an evolving encephalopathic process.

### 2.2 Relevant Medical History

The patient's past medical history was notable for nonischemic cardiomyopathy, with a previously reduced left ventricular ejection fraction (LVEF) of 30%–35%, which had improved to 55%–60% on follow‐up imaging. Her psychiatric history included diagnoses of bipolar disorder, generalized anxiety disorder (GAD), and major depressive disorder (MDD). Additional comorbidities included asthma, gastroesophageal reflux disease (GERD), and fibromyalgia. She had a history of bilateral pulmonary embolism and deep vein thrombosis, for which she was maintained on long‐term anticoagulation with apixaban.

**FIGURE 1:**
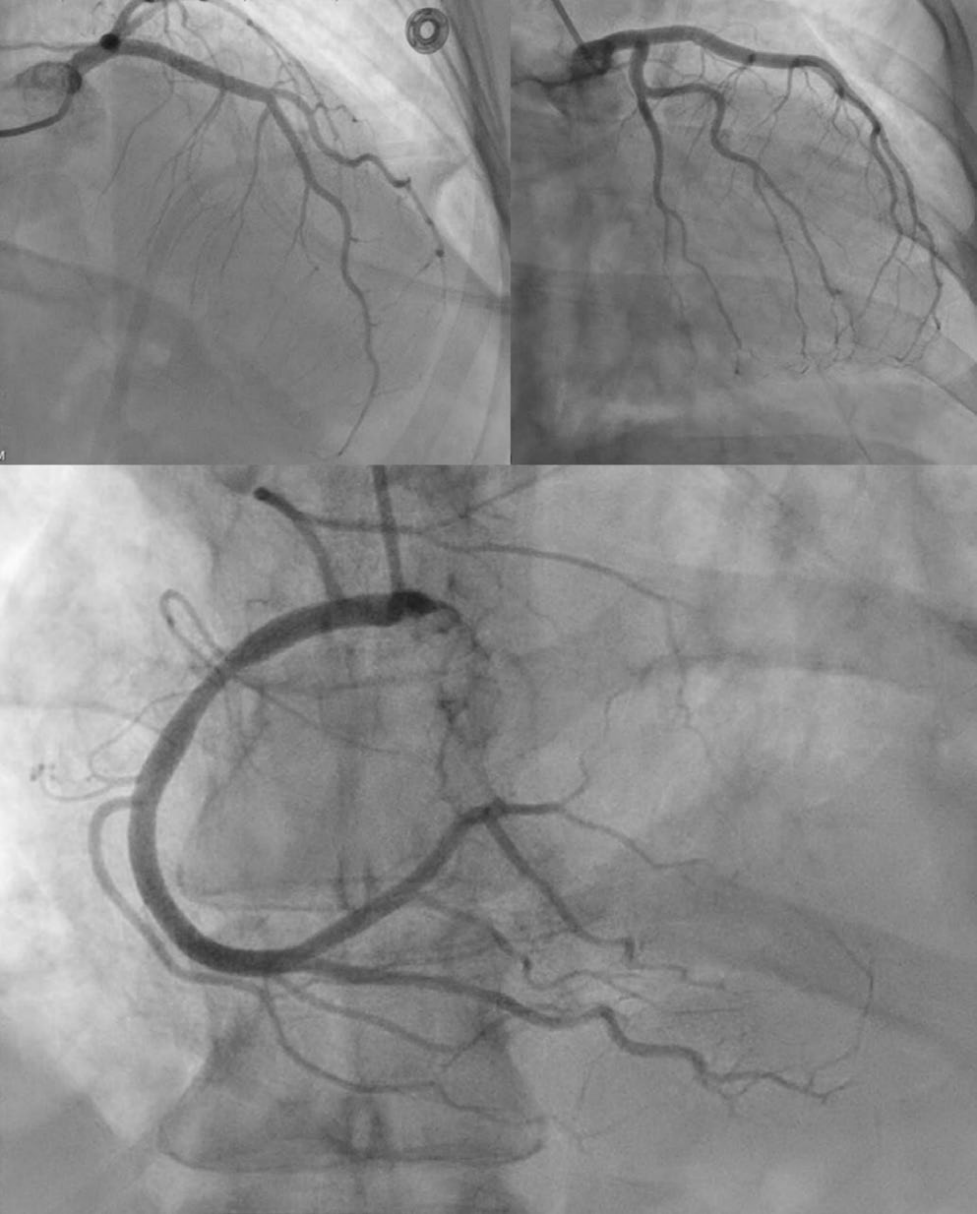
Coronary angiogram showing non obstructive coronary disease in the left (top) and the right (below) coronary arteries.

Her psychiatric medications at baseline included olanzapine, oxcarbazepine, buspirone, escitalopram, trazodone, and clonazepam. Of particular note, she had recently been hospitalized for toxic metabolic encephalopathy, non‐ST‐elevation myocardial infarction (NSTEMI), multifocal pneumonia, and acute respiratory failure requiring intubation. During that prior hospitalization, coronary angiography had demonstrated nonobstructive coronary artery disease (Figure 1), and transthoracic echocardiography had revealed a reverse Takotsubo cardiomyopathy pattern, characterized by basal hypokinesis and preserved apical function.

### 2.3 Diagnostic Evaluation

Given her acute neuropsychiatric deterioration, initial diagnostic efforts focused on excluding stroke. Non‐contrast CT and CT angiography of the head and neck revealed no evidence of large vessel occlusion. However, independent review of the imaging raised concern for a possible M2 subocclusive thrombus. CT perfusion demonstrated symmetric perfusion throughout the cerebral hemispheres, effectively ruling out acute ischemia.

A urine toxicology screen was positive only for tetrahydrocannabinol (THC), with no other substances detected. Chest radiography was unremarkable, while CT imaging of the chest ruled out pulmonary embolism but revealed a mildly enlarged pulmonary artery and a 2.3 cm pulmonary nodule in the left lower lobe. Laboratory studies demonstrated leukocytosis, a high anion gap metabolic acidosis, and an elevated lactate level of 4.6 mmol/L, which normalized after fluid resuscitation. Troponin I levels were markedly elevated, rising from 15 to 4504 ng/L over 24 h. Venous blood gas analysis showed a pH of 7.536, and urinalysis was positive for ketones, protein, and glucose. Ammonia and ethanol levels were within normal limits. Brain MRI and EEG were ordered for further evaluation of her encephalopathic presentation, and a CT scan of the abdomen and pelvis was obtained in response to persistent abdominal discomfort. Jardiance (empagliflozin) was held due to concern for possible euglycemic diabetic ketoacidosis, although this diagnosis was eventually deemed unlikely based on rapid clinical improvement following hydration.

### 2.4 Cardiac Findings and Management

**FIGURE 2:**
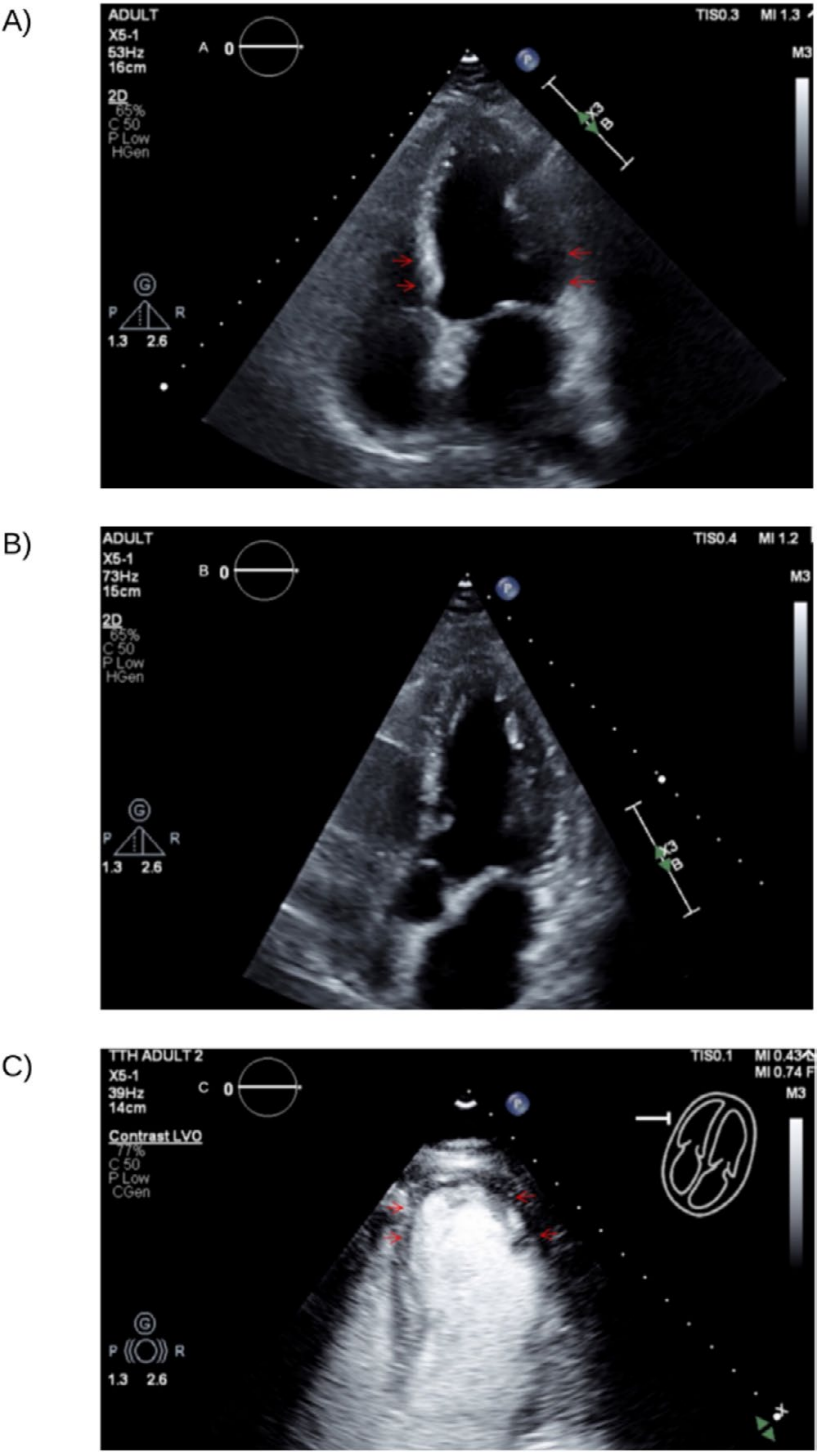
(A) Initial echocardiogram shows reverse takotsubo pattern with basal hypokinesis (red arrows) and apical sparing. (B) Subsequent echocardiogram shows the recovery of wall motion done after months. (C) Last echocardiogram demonstrates recurrence with typical takotsubo pattern of apical ballooning (red arrows) and basal hyperkinesis.

In the setting of elevated cardiac biomarkers and a known history of cardiomyopathy, cardiology consultation was obtained. Transthoracic echocardiography revealed a significantly reduced left ventricular ejection fraction of 25%–30%, with apical akinesis and evidence of swirling flow consistent with apical ballooning. These findings supported a diagnosis of Takotsubo cardiomyopathy and represented a recurrence with a different regional variant from her previous reverse pattern. (Figure 2; Video 1).

**VIDEO 1:** Transthoracic echocardiogram video clip demonstrating apical ballooning pattern consistent with Takotsubo cardiomyopathy. Video content can be viewed at https://onlinelibrary.wiley.com/doi/10.1002/ccr3.70635.

**FIGURE 3:**
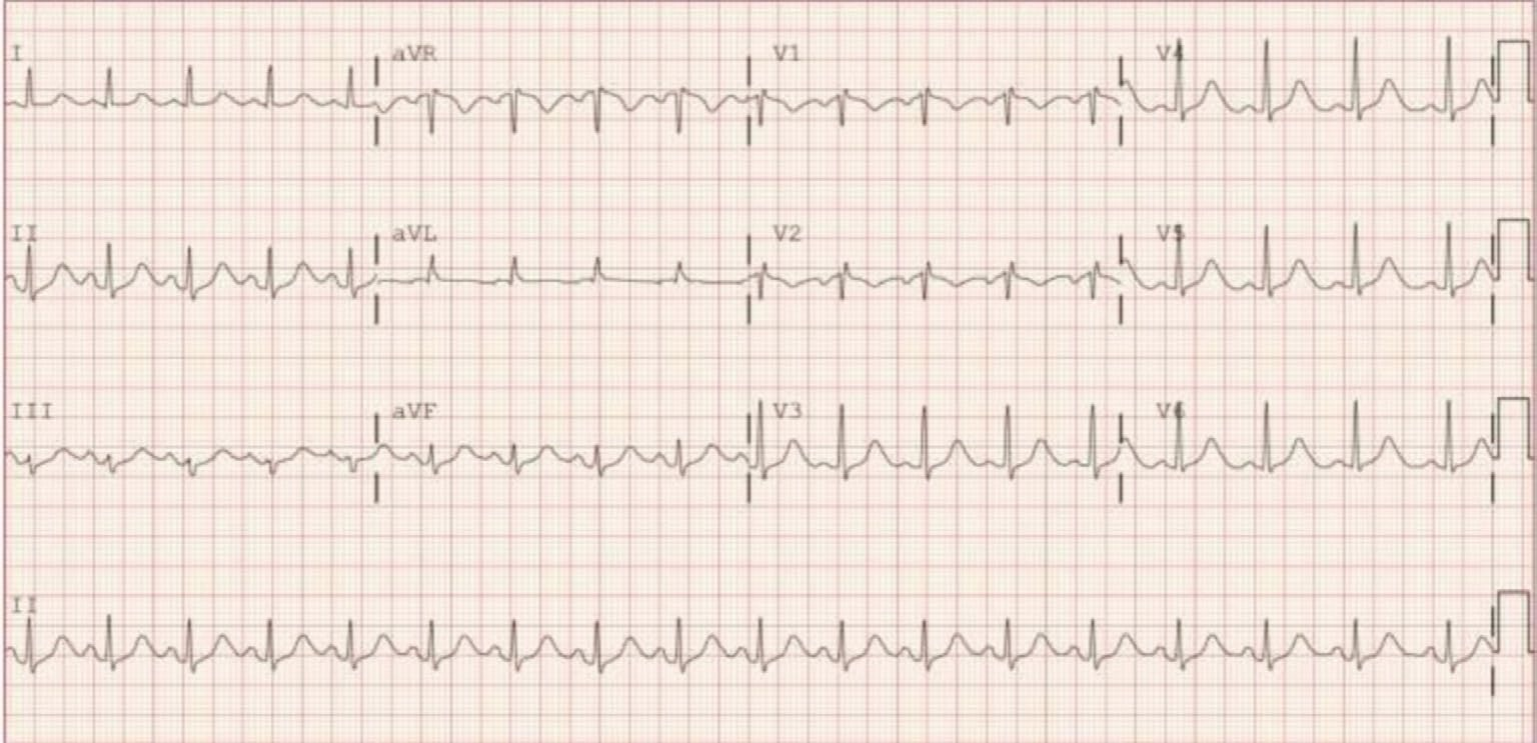
Electrocardiogram showed non‐specific ST changes and prolonged Qtc.

Electrocardiographic monitoring showed no ST‐segment elevations but did reveal QTc prolongation (Figure 3). Given the presence of apical stasis and high thrombotic risk, intravenous heparin therapy was initiated. At the time of admission, the patient was not taking her baseline psychiatric medications due to prior concerns about polypharmacy and possible medication‐related encephalopathy. Following psychiatric consultation, most psychotropic agents were temporarily withheld, oxcarbazepine was resumed, and nighttime quetiapine was started at a low dose. Delirium precautions were implemented. Neurology was consulted and, after evaluation, ruled out seizure activity despite the presence of movements suggestive of convulsive phenomena.

### 2.5 Outcome and Follow‐Up

The patient was diagnosed with recurrent Takotsubo cardiomyopathy, now presenting with an apical ballooning variant in contrast to her prior basal pattern. The recurrence was believed to be triggered by acute psychological distress and psychiatric destabilization. Her clinical status gradually improved with supportive care, anticoagulation, and selective reintroduction of psychotropic medications. Serial imaging and neurologic studies excluded underlying structural brain disease and seizure activity. Management emphasized coordinated care across cardiology and psychiatry. At discharge, follow‐up cardiac imaging and comprehensive outpatient monitoring were recommended to support long‐term recovery and minimize the risk of further episodes.

## 3 Discussion and Review of the Literature

### 3.1 Overview and Case Context

Our patient's clinical course—initially presenting with reverse (basal) Takotsubo cardiomyopathy (TTC) during a critical medical illness, followed by classical apical ballooning TTC during an acute psychiatric crisis—exemplifies both the phenotypic and etiologic variability observed in TTC recurrence. This case highlights several important themes, including recurrence patterns, psychiatric risk factors, pathophysiologic hypotheses, imaging diagnostics, and clinical management strategies. To place this case in context, we reviewed 29 previously reported cases of recurrent TTC (Table 1), providing insight into common triggers, variant switching, comorbid conditions, and recovery trajectories.

**TABLE 1:** Overview of case reports documenting recurrence and variants of Takotsubo cardiomyopathy.

Author/year	Patient demographics	Comorbidities	TTC variant(s)	Triggering event	Psychiatric involvement	Clinical presentation	Imaging findings	Treatment/management	Hospital course	Outcome	Recurrence	Unique findings/comments
Battineni et al. (2017) [8]	69‐year‐old female	Myasthenia gravis, Grave's disease, CAD, COPD	First: Mid‐ventricular, Second: Apical ballooning	Myasthenia crisis, possibly precipitated by methimazole and hyperthyroidism	Not specified	Chest pain, palpitations, ST elevation, troponin elevation	Echo: EF 25%–30%, Mid/apical hypokinesis; No CAD	Plasma exchange, steroids, IVIG (maintenance)	Resolved after PLEX both times, discharged stable	Full recovery both times, recurrence after 1 year	Yes—1 year between episodes	TTC as complication of MG crisis; co‐triggered by Grave's disease and medication
Kashou et al. (2018) [9]	59‐year‐old female	Hypertension, dyslipidemia, recurrent VF, tobacco use	Apical ballooning	Emotional stress (caregiving), possibly spontaneous	Implied (emotional stress)	Chest pain, ST elevations, troponin elevations, VF episodes	Echo: Apical/inferolateral akinesia, EF 25%–40%; No CAD	ICD placement, ACE‐I, beta‐blocker, amiodarone, other cardiac meds	Recurrent VF, catheterizations, ICD placed, stabilized	Recovered EF, long‐term ICD management	Yes, 3 episodes—Spanning over 3 years	Ventricular fibrillation requiring ICD; not a benign entity
Javed et al. (2024) [8]	61‐year‐old African American woman	HTN, DM2, gastroparesis, PE, *H. pylori* infection	Apical ballooning	Abdominal illness, emotional stress, enterovirus infection	Not specified	Chest pain, T wave inversions, troponin rise, mural thrombus	Echo: EF 35%, mural thrombus, LV hypertrophy; No CAD	Heparin, aspirin, ACE‐I, beta‐blockers, later warfarin	Renal infarct discovered, anticoagulated and improved	Stabilized, discharged with oral warfarin	Yes—2 years between episodes	Rare case with renal infarction from emboli
Carey et al. (2022) [10]	53‐year‐old woman	Chronic anxiety, HTN, hypercholesterolemia, IBS	First and third: Classical; Second: Inverted	Emotional stress (1st), none reported (others)	Yes—chronic stress and anxiety	Chest pain, ST changes, elevated troponins	Echo & CMR: classic and inverted variants; No CAD	Beta‐blocker, ACE‐I, diuretics, mental health support	Recovered fully after each event	Normal cardiac function on follow‐up	Yes, 3 episodes including rare inverted form—Over 4 years	Rare inverted form; mental health suggested as preventive target
Sahu et al. (2020) [11]	53‐year‐old woman	DM, HTN, persistent AF	Apical ballooning	First: death of parents (emotional), Second: *E. coli* sepsis (physical)	First event (emotional grief)	Dyspnea, fever, dysuria, AF with RVR, hypotension	EF 15%, apical ballooning, mild MR	IV ceftriaxone, beta‐blocker (metoprolol), ACE‐I (lisinopril)	Improved after infection treatment and cardiac meds	EF improved to 40%, stable	Yes—2 years between episodes	Triggers include both emotional and physical; sepsis is a notable cause
Vreeburg et al. (2024) [12]	All female, average age 68.6	Systemic sclerosis (SSc); various organ involvements incl. GI, renal, Raynaud's	Mixed—apical ballooning, regional wall motion abnormalities	Scleroderma renal crisis, GI involvement, physical stress, unclear	Not reported	Chest pain, ST/T changes, elevated troponin, LV dysfunction	CMR & echo: transient dysfunction, non‐ischemic fibrosis, T1 mapping, normal coronaries	ACE inhibitors, beta‐blockers	Improved with conservative therapy, recurrence noted in 2 patients	Recovery in all, monitoring advised	Yes (2 of 5 cases)—Several years (up to 7 years)	SSc may be a trigger or pathophysiologic substrate for TTS; largest SSc + TTS case series
Chandy & Dawson (2019) [13]	76‐year‐old White Scottish woman	Hypercholesterolemia, family history of CAD	Apical, apical‐mid, and focal ballooning	Bereavement, caregiving, emotional stress	Emotional stress reported	Chest pain, ST/T changes, elevated troponins, QT prolongation	Normal coronaries, CMR ruled out MI, echo/MRI showed varied TTC patterns	No long‐term meds; supportive care	Full recovery each time	Preserved EF, six episodes over 33 years	Yes (6 times)—Over 33 years	Rare long‐term tracking with phenotypic variation and multiple misdiagnoses
Ackerschott et al. (2023) [14]	51‐year‐old woman	None reported; congenital coronary anomaly (RCA from left sinus)	First: Apical; Second: Basal; Third: Midventricular	Emotional stress (work‐related)	Not formally reported	Chest pain, elevated troponin, no ECG abnormalities, elevated NT‐proBNP	CMR: Variable wall motion patterns, ischemic scar; CTA: Aberrant RCA	Beta‐blocker, ACEi, aspirin; surgery deferred	Stable recovery; medical management optimized	Good recovery; conservative management chosen	Yes (3 episodes)—Over 7 years	Coexistence of TTC and MI; first reported case with TTC and infarction in aberrant RCA
Waheed et al. (2021) [15]	72‐year‐old woman	HTN, diabetes mellitus, prior TC (8 years earlier)	Apical ballooning	Emotional stress (argument with daughter)	Emotional stress reported	Chest pain, ST elevation (I, II, aVL), elevated troponin and CK‐MB	Normal coronaries on angiography, ventriculogram and TTE showed apical ballooning; EF 21%–25%	Beta‐blocker, ACEi, statin, supportive care	Gradual improvement in EF (to 35%) and symptoms over one week	Recovered with improved LVEF and resolution of symptoms	Yes—8 years between episodes	Mimicked anterior STEMI; emphasis on careful differential diagnosis and avoidance of IABP in LVOTO
Patel et al. (2016) [16]	55‐year‐old Black woman	HTN, DM, hyperlipidemia, sarcoidosis, Graves' disease	Apical ballooning (both episodes)	First: emotional stress; Second: recurrent hyperthyroidism (no stressor)	None reported	Chest pain, dyspnea, ST/T changes, elevated troponin and CK‐MB	Echo: LVEF 30%–40% with apical/mid‐ventricular akinesis; Normal coronaries	Methimazole, furosemide, beta‐blocker, ARB; definitive radioiodine ablation	Improved after thyroid normalization; recurrence resolved after definitive treatment	EF normalized, no recurrence after euthyroid state achieved	Yes—3 years	Demonstrates causative role of hyperthyroidism; underscores need for thyroid screening in TTC
Akram et al. (2024) [17]	38‐year‐old female	Cyclical vomiting syndrome, anxiety, PTSD, long QT (KCNH2 gene), ICD	Apical ballooning, mid‐apical akinesis	Cyclical vomiting episodes	Yes—anxiety, PTSD	Chest pain, T wave inversions, elevated troponin, torsades de pointes	Echo: EF 33% (initial), 20%–24% (recurrent); apical and mid‐segment wall motion abnormalities; normal coronaries	GDMT (beta‐blocker, ACEi, MRA), colchicine, magnesium/potassium, mexiletine, psychiatric support	Recurrent torsades, defibrillation, stabilization with electrolyte correction and antiarrhythmics	EF normalized after 8 weeks; stable on follow‐up	Yes—10 months between episodes	Rare trigger (CVS), life‐threatening arrhythmia (torsades), importance of psychological care
Wu et al. (2021) [18]	55‐year‐old postmenopausal woman	History of pacemaker implantation	Apical ballooning	Repeated emotionally stressful events (quarrels, business failure)	Not formally diagnosed but emotional stress clearly linked	Chest pain, ECG changes (ST/T), elevated troponin (up to 2.228 ng/mL)	Echo: apical/mid akinesis, EF 47%–52%; Left ventriculogram confirmed apical ballooning; Normal coronaries	Perindopril and metoprolol	Stable recovery after each episode; no complications	EF improved to 55%; asymptomatic during 9‐month follow‐up	Yes (3 episodes) —3 years	Delayed diagnosis due to low awareness; importance of left ventriculogram in early recognition
Itagaki et al. (2022) [19]	51‐year‐old Japanese woman	Anorexia nervosa, hypoglycemic coma history	Basal‐type	Recurrent hypoglycemia due to anorexia nervosa	Yes—long‐term anorexia nervosa with psychiatric meds	Impaired consciousness, shock vitals, QS pattern V1–V4, elevated BNP	Echo: EF 8.6%, basal dilation, apex contraction; No CAD; recovery to EF 27.9%	ICU care, IV glucose, vasopressors (norepinephrine, vasopressin, dobutamine)	Stabilized after 10 days; discharged after 3 months	Full recovery; first documented case of TTS recurrence due to hypoglycemia	Yes – 1 year between episodes	First documented case of hypoglycemia‐triggered recurrent TTS
Tunzi et al. (2020) [20]	64‐year‐old White woman	Anxiety, no CAD, no substance abuse	Apical ballooning	Emotional stress: death of mother, family conflict	Yes—anxiety, improved with SSRI + CBT	Chest pain, ST elevation in lateral leads, elevated troponin (0.373)	Echo: EF 40%, apical hypokinesis; Normal coronaries; Typical TTC pattern	BB, ACEI, SSRI (sertraline), CBT	Recovered both times; no 3rd recurrence after SSRI+CBT	Asymptomatic on follow‐up, EF normalized	Yes (2 episodes) —1 year	Suggests SSRI and CBT may help prevent recurrence
Velasco‐Malagón et al. (2022) [21]	56‐year‐old mestizo woman from Bogotá	Hypertension, hypothyroidism	Apical ballooning	Emotional stress (unspecified)	Not diagnosed; emotional stress present	Chest pain, dyspnea, leg edema; T‐wave inversions V1–V4, high troponins	Echo: LVEF 38%, apical/mid akinesis; Angio: no CAD	Aspirin, clopidogrel, atorvastatin, enalapril, metoprolol; antiplatelets later discontinued	Stable; discharged after 4 days	EF normalized (52%) by 8 weeks, no new symptoms	Yes (after 4 years) —4 years	Emphasizes unknown recurrence risk; echoes registry recurrence patterns
Saito et al. (2023) [22]	73‐year‐old woman	Dyslipidemia	First: Apical; Second: Mid‐ventricular	Same emotional stressor (quarreling with husband)	Not diagnosed	Chest pain, ECG changes, elevated hs‐TnT & CK‐MB, distinct ECG patterns for each episode	Echo and LVG: Apical ballooning (1st), mid‐ventricular (2nd); no CAD	Carvedilol (increased dose), later added enalapril	Stable; ECG normalized by 2 weeks post‐recurrence	Full recovery after both episodes	Yes (2 episodes with different morphologies) —2 years between episodes	Demonstrates ballooning pattern shift; rare ECG variation
Saito et al. (2023) [23]	68‐year‐old woman	Dyslipidemia, oophorectomy (postmenopausal)	First and third: Mid‐ventricular; Second: Apical	Emotional stress (first), none for 2nd/3rd	Not diagnosed but emotional stress noted	Chest pain, hypertension, elevated Tn‐T	Normal coronaries; alternating echo/LVG features (apical ↔ mid‐ventricular); MRI confirmed wall motion abnormalities and edema	ACEi (enalapril), spironolactone, bisoprolol	Recovered after each episode; ECG normalized	No recurrence after β‐blocker + ACEi combo	Yes (3 within 2 months) —4–6 months	Rare intra‐month phenotypic variation; suggests estrogen and combined therapy roles
Ahmed et al. (2017) [24]	48‐year‐old woman	Postpartum depression	Apical ballooning	Recurrent severe emotional stress	Yes – emotional stress, postpartum depression	Chest pain, LOC, QT prolongation, TdP, VF	Echo: LV apical ballooning, EF 30%, LV thrombus; normal coronaries	Magnesium, mexiletine, ICD, anticoagulation	Recovered; ICD implanted for VF prevention	No further episodes; EF normalized	Yes (≥ 3 episodes over 7 years)—7 years	Rare case with TdP, VF, and apical thrombus; highlights need for aggressive management in LQT settings
Platzer et al. (2020) [25]	69‐year‐old woman	Hypertension, anxiety, pheochromocytoma (discovered later)	Multiple—apical (1st & 2nd), reverse (3rd)	Unrecognized pheochromocytoma	Anxiety disorder, beta‐blocker intolerance	Neurological signs, hypertensive crises, QT prolongation	Echo: apical and basal dysfunction; CT: adrenal mass; Labs: high plasma metanephrines	Adrenalectomy, alpha/beta blockade, supportive cardiac care	Stabilized post‐op with resolution of cardiomyopathy and anxiety	No recurrence post tumor removal; normalized LVEF	Yes (4 episodes over 3 years) – 1 year post‐adrenalectomy	Rare pheochromocytoma case with both apical and reverse TTC; delayed diagnosis
Cerrito et al. (2012) [26]	83‐year‐old woman	HTN, hyperlipidemia	Apical ballooning (both events)	Emotional stress (family quarrel; husband's death)	No formal diagnosis, but clear emotional stressor	Chest pain, ECG with ST‐elevations & T inversions, elevated troponin	LV angio: apical ballooning; normal coronaries; echo: EF drop to 35%, mitral regurgitation, transient LVOT obstruction	Conservative; monitored in hospital	ST changes and LV dysfunction resolved by Day 4	Full recovery; longest documented recurrence interval	Yes (10 years apart) – 10 years between episodes	Very late recurrence; identical ECGs and clinical pattern
Korabathina et al. (2018) [27]	Patient 1: 67F; Patient 2: 75F	COPD, gastritis, cholecystitis	Pt 1: All midventricular; Pt 2: Apical then 2× Basal	Various medical stressors	Not reported	ECG: Deep T inversions, ST depressions; elevated troponin, BNP	Echo/LVG showed ballooning in changing wall segments; all had preserved coronaries	β‐blockers, ACEi (where tolerated); Pt 2 no HF therapy due to hypotension	All resolved within 1–3 months	EF normalized in all events	Yes (3 events per patient) – 1–2 years	Both patients showed different ballooning morphologies in recurrence
Ahmadjee et al. (2020) [5]	75‐year‐old woman	Diabetes mellitus, HTN, hyperlipidemia, COPD, CKD Stage 3	First: Classical (apical); Second: Reverse (basal)	Both triggered by emotional stress	None diagnosed; both events preceded by emotional stress	Chest pain, mild ST‐T changes, elevated troponin	Echo: Apical akinesia (1st); basal hypokinesia with apical hyperkinesia (2nd); normal coronaries both times	Beta‐blocker, ACEi, statin, diuretic	Recovered fully after each episode	EF normalized to 60% after second event; longer recovery time noted in 2nd recurrence	Yes (2 variants, 1.5 years apart) —7 months post‐2nd event	Rare presentation of both classical and reverse variants in same patient
Mainali et al. (2013) [28]	51‐year‐old woman	SLE, hypothyroidism, prior Takotsubo cardiomyopathy	Apical ballooning (recurrent)	Emotional stress (death of a close friend)	Yes—emotional grief	Chest pain, SOB, nausea, T wave inversions, troponin rise	Echo: EF 43% with apical hypokinesis; previous episode: EF 25%	Aspirin, ACE inhibitor, beta‐blocker	Stable recovery; EF normalized to 61% post‐discharge	Full recovery both episodes	Yes —2 years between episodes	Recurrent TTC induced by intense emotional stress; apical pattern
Sager et al. (2011) [29]	66‐year‐old woman	COPD, peripheral arterial disease (aortobifemoral bypass)	Mid‐ventricular (same variant all 3 episodes)	COPD exacerbation, family dispute, arterial occlusion	Emotional and physical stress	Chest pain, ST changes, elevated TropT, T wave inversions	Angiography: hypokinesis of anterior/basal segments sparing apex	Supportive care, antiobstructive therapy	Recovery of LV function within 1–2 weeks post each episode	Full recovery after each recurrence	Yes, 3 episodes—Over 4 years	Rare case with consistent mid‐ventricular pattern despite varying stressors
Wever‐Pinzon et al. (2010) [30]	82‐year‐old woman	Hypertension	First: Mid‐ventricular; Second: Apical + mid	Emotional stress (relative's death, unspecified event)	Emotional stress	Chest pain, pulmonary edema, ECG ST elevations, mild troponin rise	Echo: Akinesis in mid/apical segments, hyperkinetic base; normal coronaries	ACE inhibitor, beta‐blocker, aspirin	Symptoms resolved within days, EF normalized	Recovered systolic function post each event	Yes, 2 episodes – 6 months between episodes	Different morphologic TTC patterns in same patient within 4 months
Bridgman et al. (2011) [31]	76‐year‐old woman	Moderate coronary artery disease (mid LAD lesion, stented)	First: Apical; Second: Mid‐ventricular (apical sparing)	Two separate major earthquakes in Christchurch (2010, 2011)	Yes—earthquake‐induced emotional trauma	Chest pain, QT prolongation, T wave inversions, SOB	First: Classic apical ballooning; Second: Mid‐ventricular variant	Aspirin, clopidogrel, simvastatin, diltiazem	Recovered well after both events; no beta‐blockers used	Full recovery with normal echocardiography post‐discharge	Yes—5 months between episodes	Rare case with different TTC variants due to same type of trigger (earthquake)
Janus et al. (2020) [32]	67‐year‐old woman	None reported	Three: Mid‐ventricular, Apical, Basal (within 3 months)	Neurological stress, hypotension, and hypoxia from aspiration pneumonia	Not specified, but stress‐induced pattern clear	ST elevations, deep T‐wave inversions, cardiogenic shock	Echocardiography: all three TTC variants observed	Supportive care; vasopressors used during hypotension	Each episode resolved within days; full cardiac recovery each time	Recovered after each variant; discharged to rehab	Yes—3 episodes within 3 months	Only reported case with all 3 TTC variants in a single patient
Pathak et al. (2010) [33]	83‐year‐old woman	Hypertension, hypercholesterolemia	Apical ballooning (both episodes)	Mild physical exertion: biking, brisk walking	Not specified	Chest pain, SOB, ST elevations, anterior/lateral leads	EF 25%–30%, apical akinesis, normal coronaries	Conservative management	Both episodes resolved fully with normalization of EF	Full recovery in both episodes	Yes—4 years between episodes	Recurrence after mild stress suggests high intrinsic vulnerability

### 3.2 Recurrence Patterns and Morphologic Variants

While TTC most often presents with apical ballooning and basal hyperkinesis, recognized variants include mid‐ventricular, basal (reverse), and focal types [34]. Recurrences commonly involve the same pattern; however, as seen in our case, variant switching does occur. In a series of nine patients with ≥ 2 recurrences, 44% experienced a change in wall‐motion abnormality distribution [35]. Our patient's presentation—apical variant after a reverse episode—adds to this growing body of literature suggesting dynamic regional involvement rather than fixed myocardial susceptibility.

Trigger variability also characterizes recurrence. Emotional stress is the most frequent precipitant, but physical stress, neurologic insults, and in some cases, trivial triggers like caffeine have been reported [4]. One analysis found that 67% of patients with recurrent TTC had different types of triggers in each episode [35]. Our patient exemplifies this: her first episode followed critical illness, while the second was triggered by psychological distress. These findings emphasize that TTC recurrence reflects an evolving interplay between stressor types and intrinsic neurocardiac reactivity.

### 3.3 Epidemiologic Insights From Literature

Consistent with TTC demographics, most recurrent cases occurred in postmenopausal women [2, 36]. Though the average age is mid‐60s, recurrences in younger women—like our 50‐year‐old, perimenopausal patient—are well documented, particularly when psychiatric comorbidity is present. Recurrence intervals vary widely. For example, Arcari et al. reported a median recurrence interval of 4.4 years [37], whereas other studies have documented recurrences within months [38, 39]. Our patient's recurrence occurred within 1 month, aligning with literature describing short‐interval “super recurrences” [35].

### 3.4 Psychiatric Comorbidity and Recurrence Risk

Psychiatric illness is a prominent risk factor for both initial and recurrent TTC. Up to 50% of patients have a psychiatric or neurologic diagnosis [36, 40]. In a systematic review, depression (39%), anxiety (17%), and serious mental illness (10%) were commonly reported [40, 41]. Psychoactive medication use is more frequent among recurrent TTC patients, reflecting this burden [42, 43]. Our patient had multiple diagnoses (bipolar disorder, generalized anxiety disorder, major depressive disorder) and was off her full psychiatric regimen at the time of recurrence—a likely contributor. This supports growing recognition of the brain‐heart axis in TTC, where emotional distress can precipitate myocardial stunning.

Other associated risk factors include COPD and diabetes, which may augment catecholaminergic drive and sympathetic tone [44]. Interestingly, while men represent only ~10% of TTC cases, they appear at a higher risk of adverse outcomes or recurrence when affected, possibly due to a higher comorbidity burden [44, 45, 46].

### 3.5 Pathophysiologic Mechanisms

TTC is believed to arise from acute catecholamine surges in response to stress. This stimulates cardiac sympathetic nerve terminals and circulating epinephrine, leading to transient myocardial stunning via β‐adrenergic receptor signaling shifts [47, 48]. The apical myocardium, dense with β‐receptors, is particularly vulnerable—explaining the classical pattern of apical ballooning.

However, variant switching, as seen in our case, suggests more complex mechanisms. One hypothesis is that different stressor types preferentially affect different myocardial zones: neurologic insults may provoke basal hypokinesis via direct cardiac sympathetic discharge, while emotional distress may trigger apical dysfunction through systemic catecholamine release [23]. Microvascular dysfunction and vasospasm may also contribute by impairing myocardial perfusion during stress [49]. Additionally, estrogen deficiency in postmenopausal women may increase susceptibility. Estrogen modulates endothelial function and blunts sympathetic activation. Its decline could lower the threshold for TTC [50, 51]. Our patient's perimenopausal status may have compounded her risk during psychological stress [52].

### 3.6 Imaging Findings and Clinical Management

TTC diagnosis requires ruling out acute coronary syndromes through coronary angiography or high‐quality noninvasive imaging [3]. In both of our patients' episodes, angiography revealed non‐obstructive disease, supporting TTC over infarction [36].

Echocardiography remains the diagnostic cornerstone, identifying characteristic wall‐motion abnormalities that extend beyond a single coronary territory [53]. In our case, apical ballooning and swirling flow were observed. Cardiac MRI (CMR) can further delineate TTC, typically showing myocardial edema without fibrosis—helpful in excluding myocarditis or infarction [54, 55].

Complications must be anticipated. Our patient had apical stasis with thrombotic risk and was treated with anticoagulation—a strategy supported by case reports of embolic events in similar presentations [8]. Other risks include arrhythmias, QTc prolongation, and cardiogenic shock [2, 36, 38].

Long‐term management often includes beta‐blockers and ACE inhibitors, though evidence on recurrence prevention is mixed [56, 57]. Observational data suggest ACE inhibitors or ARBs may be more effective than beta‐blockers alone in reducing recurrence [58, 59]. Our patient's regimen was updated accordingly. However, the cornerstone of TTC prevention remains trigger mitigation—especially emotional stressors—underscoring the need for psychiatric stabilization and follow‐up.

### 3.7 Case Integration and Teaching Points

Our case contributes to the literature in several unique ways:

1. Phenotypic switching: The patient exhibited distinct TTC variants in close succession, reinforcing that regional myocardial susceptibility is dynamic, not fixed.
2. Psychiatric trigger: Acute psychiatric decompensation was the clear precipitant of the second episode, emphasizing the central role of emotional health.
3. Recovery and follow‐up: Complete recovery of systolic function was achieved between episodes, consistent with TTC's generally favorable prognosis, though recurrence risk remains.

Multidisciplinary care—including cardiology, psychiatry, neurology, and critical care—was crucial in her outcome. This supports recommendations for comprehensive care models in TTC, particularly in patients with psychiatric comorbidity or recurrence risk. Patients should be educated on the signs of recurrence, the impact of stress, and the importance of adherence to psychiatric and cardiac therapy.

With appropriate management, the prognosis for TTC remains excellent. Acute‐phase mortality is low (~1%–2%), and most patients regain full cardiac function [36]. However, recurrence is not rare, and vigilance is warranted—especially in younger women with emotional or psychiatric vulnerabilities.

## 4 Conclusion

This case underscores the complex and heterogeneous nature of Takotsubo cardiomyopathy, particularly in recurrent presentations with variable morphological patterns. The dual episodes—reverse and classic variants—highlight how different stressors, especially psychiatric triggers, may influence regional cardiac responses. Psychiatric comorbidities, as demonstrated, play a pivotal role not only in the pathogenesis but also in recurrence risk, emphasizing the need for integrated care. Long‐term prognosis remains favorable with appropriate management, but recurrence vigilance and multidisciplinary collaboration, particularly between cardiology and psychiatry, are essential to optimize outcomes and prevent future episodes.

## Author Contributions

**Hamza AlKowatli:** conceptualization, supervision, writing – original draft, writing – review and editing. **Oubada Alkowatli:** conceptualization, writing – original draft, writing – review and editing. **Ahmad Karzoun:** methodology, writing – original draft, writing – review and editing. **Bisher Sawaf:** conceptualization, methodology, supervision, writing – original draft, writing – review and editing. **Mhd Baraa Habib:** methodology, resources, writing – original draft, writing – review and editing. **Nahush Bansal:** conceptualization, writing – original draft, writing – review and editing. **Muhammad Faiz:** methodology, resources, writing – original draft, writing – review and editing. **Robert Subbiondo:** supervision, writing – review and editing.

## Consent

Written informed consent was obtained from the patient for anonymized patient information to be published in this article.

## Conflicts of Interest

The authors declare no conflicts of interest.

## Data Availability

The datasets generated during and/or analyzed during the current study are available from the corresponding author upon reasonable request.
